# Heigham Hall Lunatic Asylum, Norwich

**Published:** 1855-01-01

**Authors:** 


### HEIGHAM HALL LUNATIC ASYLUM, NORWICH.

It is so seldom that the public mind is ill directed or unfairly prejudiced by the public press, or that the latter appears to lend its aid to attack the innocent or minister to vindictive and personal feelings, that whenever such an event does occur, the attention of thinking and unprejudiced men is arrested, and the mind seeks some explanation of such an apparent anomaly.

There are few subjects which have of late occupied earnest and philanthropic minds more than the management of asylums for the insane, and the treatment of their inmates; nor is there any more vexed question than the boundary which separates soundness of mind from insanity.

The very fact of mental disease requiring peculiar treatment, and that for obvious reasons such treatment is at all times attended with a certain amount of secresy, and that the interior of a private asylum should be as private as its name designates, gives rise in the minds of the public to a certain feeling of curiosity, not unattended by doubt and suspicion. Hence any apparent departure from the laws which govern such institutions is watched with jealous scrutiny. The antecedents of private asylums before the appointment of Commissioners in Lunacy are not of the most favourable kind; while the supervision of that body has not been long enough established, nor their peculiar functions sufficiently understood by the public, to give them the assurance that the misrule of former days is now practically impossible. Hence it is, we believe, that a whisper of mismanagement in any public or private asylum is eagerly listened to; and minds already prepared for startling disclosures seize with avidity upon the first intimation of illegal practices, and at once draw the conclusion that heinous offences can be and still are perpetrated and sheltered under the existing laws of lunacy.

Time alone can give a different bias to public opinion upon this subject; and it is not until the public mind is satisfied that, by the machinery of the lunacy laws, a watchful eye is ever kept on the conduct of asylums, and that misdeeds are inevitably detected and as surely punished, that mistrust of such establishments will be removed.

We have been led to make the above remarks by the report of a recent case which has excited no little public interest, and not less public scandal—a case which, if the facts are as represented by the public statements, should for ever deprive the parties accused of all public confidence; and it is not without much pain, seeing that gentlemen of high and rising repute in their profession are implicated as to character and reputation, that we commence the investigation of this case. There is in the city of Norwich a private lunatic asylum, known as Heigham Hall, which for more than twenty years has been conducted with repute by its proprietors. Its position was so good that during the past year Dr. Ranking, a gentleman well and most favourably known in medical literature, as well as highly esteemed in his immediate neighbourhood, was induced to become a co-proprietor with Messrs. Nichols and Watson, the original proprietors. Immediately after this union reports were circulated by Dr. Hull, that, *two years* before, Mr. Nichols had solicited him to become a party to a breach of the law. Now, it is not our intention to discuss the private squabbles of Mr. Nichols or Dr. Hull, or entertain the question of the motives of the one or the credibility of the other. We have a higher and more serious object in view. We desire to enter dispassionately into the inquiry—seeking the truth, and awarding an unprejudiced judgment. The allegations against Messrs. Nichols and Watson may be considered under three heads:—

*Firstly.* That they, in June, 1852, received into their house a person guilty of an offence, of whose insanity there was doubt, and by such reception rescued him from the probable consequences of his crime.

*Secondly.* That upon his discharge he was appointed chaplain to the asylum.

*Thirdly.* That after the visiting justices, in June, 1854, made the discovery that, in their opinion, he was not a proper person to have been appointed, or continue to officiate, as chaplain, he was, in contravention of the Lunacy Act, retained in the house as a boarder. These, apart from a vast deal of personal and extraneous matter, constitute the gravamen of the charges against the proprietors.

Let us now investigate the first, and by far the most serious charge:—Was Mr. Holmes (the patient) sane or insane?—and was he protected, by the admission to the asylum, from the operations of the law?

It appears that his family, on a previous occasion, entertained doubts as to his sanity, and, at that time, Mr. Nichols had an opportunity of adding a patient to his establishment, but declined, because, in his opinion, the *evidence of insanity was insufficient,* a line of conduct not in accordance with the charges recently brought against him: being again consulted, he deemed him of unsound mind, and advised his confinement; and, in this opinion, he appears supported by the testimony of all the parties concerned in the transaction, as is apparent from the statements of the rector of the parish, the magistrate who was applied to, the policeman who investigated the charge, the mother of the girl upon whom the assault was committed, her aunt, Mr. Holmes' housekeeper, and her husband, who all came to the same conclusion, that he was not and had not been for some time in his right mind before his removal to the asylum. On the other hand, a Mr. Mills, who had been requested to examine Mr. H——, could not discover any symptoms of insanity (he does not say that he was of sound mind), and upon this point much stress has been laid. With us it has but little importance; for it must be considered that Mr. Mills saw the patient but *once;* now there are many cases in which a single interview is sufficient to detect unsoundness, but it not unfrequently occurs that several examinations are required before the experienced and practical psychologist can venture to pronounce upon the existence of insanity, and we cannot help thinking that the evidence of those in daily intercourse with Mr. Holmes is more than sufficient to counterbalance the negative opinion of Mr. Mills. It is stated, upon credible testimony, that this unfortunate gentleman expressed to the Rev. Mr. Andrew that it was his desire to be placed in the Norwich Castle, as that would do him good. The husband of this housekeeper states, that he had, on many occasions, to hold him down on the floor as a protection from his violence; and the general evidence of other parties is to the effect that his demeanour was that of a lunatic. The Rev. Mr. Cobb says, that, "during his sojourn at Wymondham, he became less and less responsible for his actions." In the face of such an amount of testimony, can we draw any other conclusion than that the mind of Mr. Holmes was upset; the contrary would be to suppose that a vast conspiracy existed to establish a fact which no one beyond Mr. Nichols had any interest in making apparent. The fact that the accused have again and again made efforts to be put upon their defence, should have its due consideration; at present they appear, owing to feelings adverted to in the earlier portion of this article, to have been unfairly assailed, and condemned unheard. The charge that Mr. Holmes was rescued from the gripe of the law involves a simple absurdity, as any one acquainted with English law must know; his admission to the asylum in no way protected him, but rather secured his person until the ministers of the law had prepared to seize him: had such a course been adopted, Mr. Nichols would have found to his cost that any opposition would have been followed by fatal consequences to his asylum. We cannot help thinking that some undercurrent prejudicial to the interests of the asylum exists, since, even after the expressed opinion of the Commissioners in Lunacy that the Rev. E. Holmes was insane when admitted to Heigham Hall Asylum, the public mind is disturbed by frequent returns to the subject. Our space prevents our entering more than generally upon the other and more insignificant charges. The propriety of the appointment as chaplain is a matter of opinion only; there was nothing morally or legally wrong in such appointment, nor can we see that a lunatic restored to reason should be held responsible, and punished for acts committed while of unsound mind, or precluded from a return to the duties of his profession.

The breach of the Lunacy Act, upon which the Recorder of Norwich made, we think, much too severe remarks, appears to us no breach at all. Mr. Holmes was suspended from his office by the proprietors; they were anxious (and diligently sought) for further investigation into the charges against them. During this time Mr. Holmes, as was most natural, remained, with the knowledge of all parties, in the house, which he at once left when the Commissioners found themselves unable to sanction his residence as boarder. The published correspondence of the Commissioners upon this point must disarm all feeling of prejudice. We have now a more pleasing duty to discharge towards the proprietors of Heigham Hall Asylum. We observe that an active magistrate, Mr. Guttren, speaks of the management of the asylum in terms of unqualified praise, an occurrence studiously overlooked in the various articles which have appeared to the prejudice of the proprietary. We are unable to account, upon reasonable grounds, for this omission. We, however, must congratulate Messrs. Nichols, Ranking, and Watson, upon having obtained, at such a time, so unqualified a commendation of their management. Let them but persevere in such a course, and we hesitate not to say they will outlive the attacks of the evil-disposed, or the attempts of unsuccessful rivals to injure an establishment which now, the first time for twenty years, has had a breath of scandal wafted against its walls.

---

